# Shear strength dataset of hollow concrete block masonry with different mortar bedding

**DOI:** 10.1016/j.dib.2024.111144

**Published:** 2024-11-19

**Authors:** Milena Mesa-Lavista, Paola Romo-Letechipía, José Álvarez-Pérez, Ricardo González-Alcorta, Jorge H. Chávez-Gómez, G. Fajardo-San Miguel

**Affiliations:** a Facultad de Ingeniería Civil (FIC), Departamento de Estructuras, Universidad Autónoma de Nuevo León (UANL), *Av*. Universidad, s/n CP. 66455, San Nicolás de los Garza, Nuevo León, Mexico; b Facultad de Ingeniería Civil (FIC). Subdirección de Posgrado e Investigación, Universidad Autónoma de Nuevo León, *Av*. Universidad, s/n CP. 66455, San Nicolás de los Garza, Nuevo León, Mexico

**Keywords:** Face-shell mortar bedding, Full-shell mortar bedding, Shear test results, Masonry wallettes

## Abstract

Masonry is a construction material composed of units (blocks or bricks) joined with mortar. It is one of the most widely used materials in construction resisting both vertical and horizontal forces in single and multi-family housing buildings. A correct union between the units and the mortar (interface) is essential, as is determining the resistance from the applied loads. There is a divergence in how the mortar is bedded in the construction of masonry walls. In some countries, such as Canada and Australia, regulations require that the mortar be placed in face shell bedding when hollow blocks are used. However, in countries like Mexico, regulations establish that it be placed in the net area, and construction practices often differ. Much research has been conducted to study the compressive behavior of mortar bedding in masonry of hollow concrete blocks. However, fewer studies have focused on shear behavior. This paper presents the dataset of experimental laboratory tests on wallettes built with hollow concrete blocks. Two methods of mortar bedding were employed: over the net area and the lateral face. The values obtained can be used to compare the shear strength in hollow concrete block masonry and the shear failure. Additionally, they can be useful for calibrating numerical models.

Specifications Table

**Table:** 

Subject	Civil and Structural Engineering.
Specific subject area	Strength shear in masonry of hollow concrete blocks
Type of data	Tables and Images in excel sheets Folder with photos
Data collection	For data collection, a data acquisition unit NI PXIe-1073 along with an NI TB-4330 Bridge Input Card and an NI TB-4340 LVDT Card from National Instruments were usedThe NI PXIe-1073 data acquisition unit has the following characteristic: • Chassis type: PXI Express • Number of slots: 9 slots (one dedicated to the controller and eight hybrid slots for PXI and PXI Express peripheral modules) • Slot size: Compatible with 3 U modules • Bandwidth: 4 GB/s (max.) • System interface: PXI Express Gen 2 with support for PCI Express bandwidth • Power supply: Universal AC power supply (100–240 V AC) • Operating temperature: 0 to 50 °C • Heat dissipation: Up to 58 W per slot • Dimensions: 177 × 214 × 211 mm • Weight: 6.55 kg • Additional features: • Forced air cooling with adjustable fans • Compact and robust chassis The NI TB-4330 Bridge Input Card has the following characteristic: • Module type: Terminal block for bridge signal conditioning modules (strain gauges) • Inputs: 8 differential input channels • Sensor type: Strain gauges • Bridge configurations: Full, half, and quarter bridge • Measurement range: ±25 mV/V to ±300 mV/V (adjustable) • Sampling rate: Up to 25.6 kS/s per channel • Accuracy: Depending on the configuration, it can achieve up to 0.05 % accuracy • Additional features: • Adjustable internal excitation up to 10 V • Thermal compensation for measurements • Integrated terminal block connector • Support for self-calibration and digital filtering The NI TB-4340 LVDT Card has the following characteristic: • Module type: Terminal block for LVDT and RVDT sensors (displacement transducers) • Inputs: 8 differential input channels • Sensor type: LVDT (Linear Variable Differential Transformer) and RVDT (Rotary Variable Differential Transformer) • Carrier frequency: Up to 25 kHz • Displacement range: Depending on the connected transducer, it can measure displacements from microns to several centimeters • Excitation: Adjustable constant current excitation output • Sampling rate: Up to 25.6 kS/s per channel • Accuracy: High precision with 50/60 Hz noise rejection • Additional features: • Support for self-calibration • Integrated terminal block connectors • Adjustable filters to improve measurement signal in noisy environments
	In addition, strain gauges, Linear Variable Differential Transformers (LVDT), and a load cell were employed. This instrumentation was used to acquire the strain, displacement, and load data while three-course hollow concrete block wallettes were tested. For testing, a load frame was built to match the dimensions of the wallettes. Shear strength was assessed using a Enerpac 100-ton hydraulic jack, model RR10013, 13 inches stroke of the hollow piston to apply loads until failure *.*
Data source location	Institution: Universidad Autónoma de Nuevo León, Instituto de Ingeniería Civil. City: San Nicolás de los Garza Town: Ciudad Universitaria Region: Nuevo León Country: Mexico (25°44′00.07′’ N, 100°18′22.55′’ W)*.*
Data accessibility	Repository name: Mendeley data Data identification number: 10.17632/9g28c874n2.1 Direct URL to data: https://data.mendeley.com/datasets/9g28c874n2/1

## 1 Value of the Data

• *Design codes:* Researchers can refine shear design codes for masonry construction, by analyzing the different mortar bedding methods for updating design codes to reflect best practices.
• *Theoretical Models:* The data can support development or improvement of theoretical models of shear behavior. It helps to improve the prediction of shear strength and failure mechanisms, advancing the structural engineering knowledge.
• *Benchmark for New Experimental Techniques:* The dataset validates new experimental methods. Researchers can compare their results with this data to ensure accuracy and reliability when testing masonry properties.
• *Educational Resource:* The data enriches structural engineering education. Educators can create case studies and exercises, enhancing students' understanding of shear strength in masonry.
• *Numerical Model Calibration:* Experimental data is key for calibrating numerical models, ensuring accurate simulations of masonry structures under different loads.

## 2 Background

**Fig. 1:**
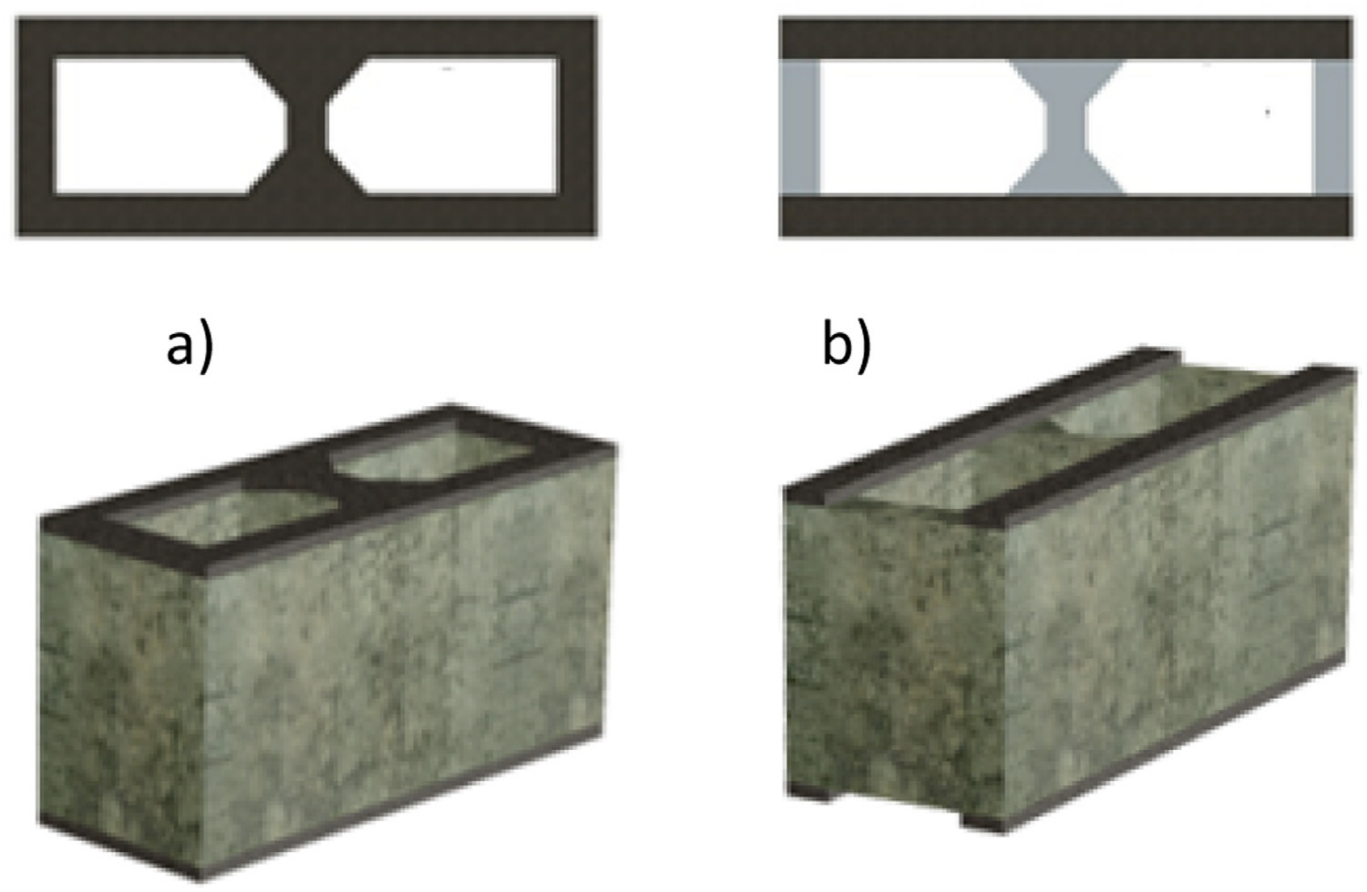
Definition of mortar bedding areas (black colored areas): a) Full mortar bedding (net area) and, b) face-shell mortar bedding (lateral areas).

**Fig. 2:**
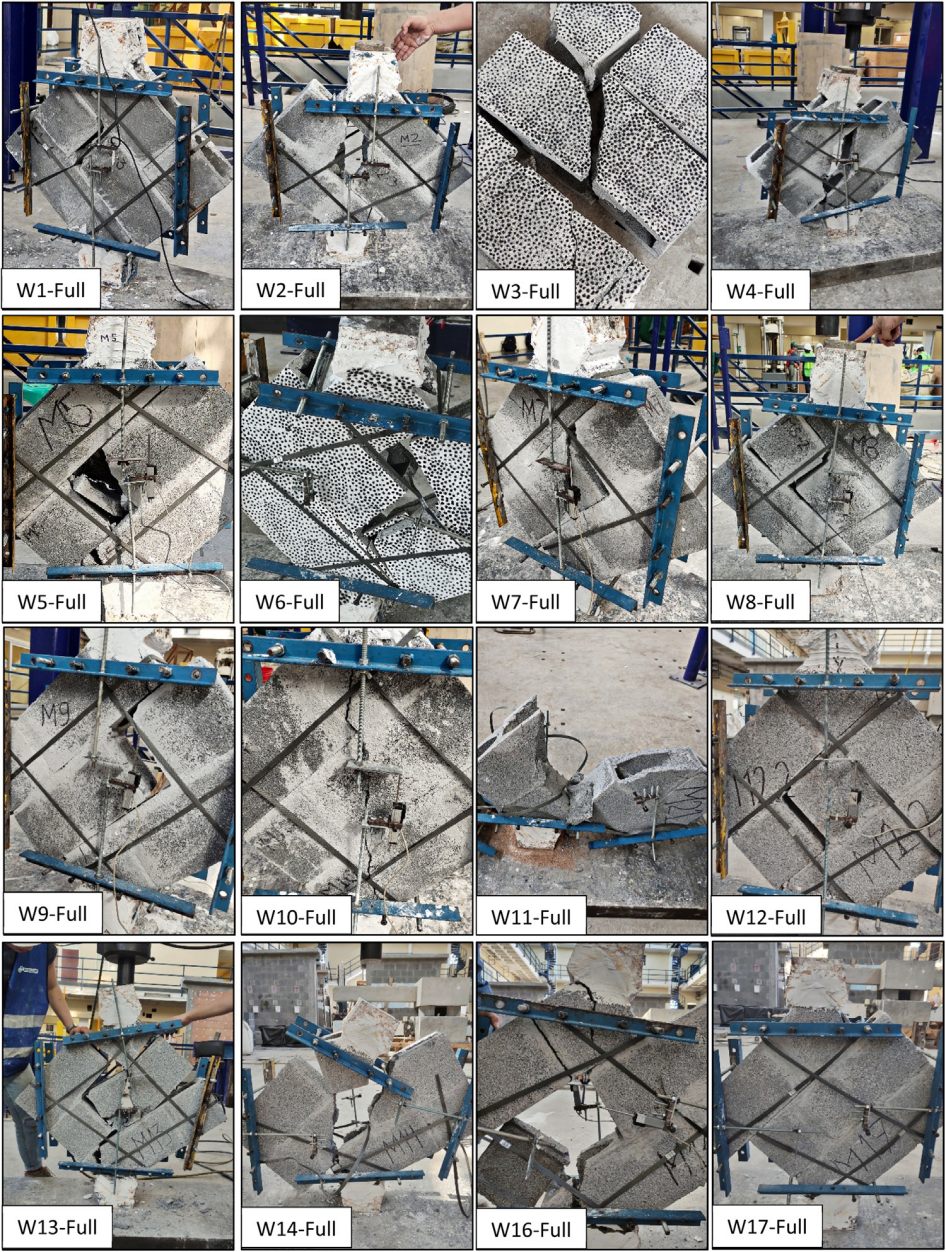
Failure in the wallettes tested under diagonal compression with full mortar bedding.

Masonry is a composite material of units joined with or without mortar [1,2]. A correct union between the units determines how the masonry transfers and resists stresses due to applied loads [2]. In the specific case of hollow concrete block masonry, there are divided views regarding the criteria for the way mortar bedding must be applied to achieve an effective interface (Fig. 1). Fig. 1 shows the two different mortar bedding used in this research: (1) applied over the entire net area of the block or full-shell bedding (Fig. 1a) and (2) applied only to the outer edges or lateral faces of the block of face-shell bedding (Fig. 1b). These methods differ primarily in their mechanical response. Full-bed bedding provides a more uniform distribution of load, while face-shell bedding concentrates the load along the edges, which can result in different failure mechanisms and overall strength [[3], [4], [5]].

Canadian [6,7] and Australian [8,9] standards indicate that mortar should be placed in face shell bedding when the blocks are hollow. While, Mexican [[10], [11], [12]] and American [13,14] standards indicate it should be placed on the net area. The European standard [15] does not make a distinction.

In Mexico, regardless of the standards indicating that the mortar should be placed on the net area, in construction practice it is increasingly placed in the face shell bedding. This changes the way masonry behaves. The compressive behavior of masonry, influenced by the mortar bedding placement, has been studied previously [16,17]. Several studies [18,19] performed compressive experimental tests on two-course concrete prisms with face-shell bedding. During failure, they noted the development of tensile stresses on the lateral faces due to the rotation and crushing of the mortar. This failure mechanism resembles that of beams, where lateral mortar crushing induces horizontal stresses, leading to bending failure of the block [19]. A similar failure pattern was recently observed in three-course prisms under compression test [5,20]. According to researchers [20], prisms with fully bedded mortar showed greater strength (peak stress) than those with face-shell bedding, which they attributed to increased tension in the web shells. However, there is an ongoing debate in the literature regarding whether face-shell or full bedding mortar provides higher compressive strength in HCB masonry.

On the other hand, the shear behavior under this variation is less studied. Moreover, there are few experimental reports analyzing the shear behavior of HCB masonry using the face-shell bedding method and comparing it with the full bed mortar technique [21,22]. Key mechanical parameters such as: 1) shear modulus (*G_m_*), 2) shear strength (*τ_m_*), and 3) ultimate shear strain (*γ_m_*) are significantly influenced by the mortar bedding method. In professional practice, the shear strength (*τ_m_*) and shear modulus (*G_m_*) are the most used parameters for evaluating the shear properties of HCB masonry. Therefore, the purpose of this research, along with the experimentation and data collection conducted, is based on the need to research the shear behavior of masonry when the mortar is placed either on the face shell or the net area of the block.

## 3 Data Description

**Fig. 3:**
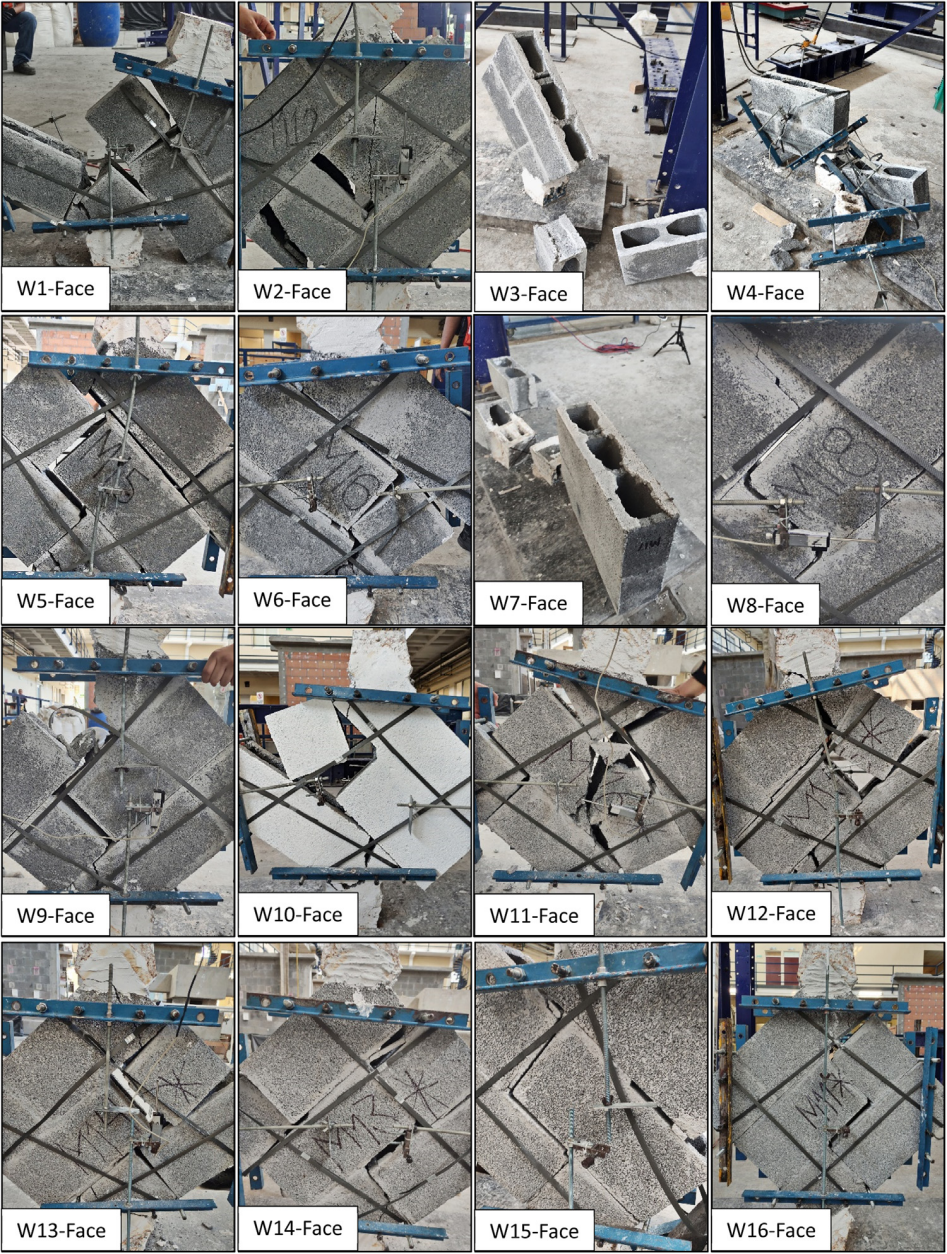
Failure in the wallettes tested under diagonal compression with face shell mortar bedding.

This paper describes the dataset from 33 wallettes tested under diagonal compression, 17 of them with mortar bedding in the net area (full) and the other 16 in face-shell bedding, as shown in Fig. 2, Fig. 3, respectively. There are no photos available for wallette number 15 (full bedding mortar). The dataset includes 32 folders, containing a photo collection from each test. Additionally, there are two Excel sheets with all the curated and filtered data. One of the Excel sheets has information about the mortar and block characterization, used in the construction of wallettes. The other Excel Sheet shows the shear stress vs. angular strain curves for each wallette test, except for M3-Full, M8-Full, and M7-Face, where Linear Variable Differential Transformer (LVDT) data could not be measured.

Table 1 shows a summary that was made also in Excel. It presents the mean and standard deviation from the wallettes tested, with both mortar bedding types. While the paper includes mean and standard deviation values, additional statistical analyses can indeed be applied. A parametric test, such as a *t*-test, or a nonparametric test, like the Mann-Whitney U test, could be used to compare the shear strength or Shear Modulus between the full and face-shell bedding types. These tests would determine whether the observed differences are statistically significant, offering deeper insights into the variance and equality of means between the two datasets. On the other hand, the table also indicates the failure recorded from these tests, being:

- ***A*** →Adherence failure: the adhesion between the block and the mortar fails, causing the joint to detach without a crack developing in the blocks. However, there may be a fracture in the piece due to tension. See the W9 in Fig. 2.
- ***DT*** → Diagonal tensile failure: a crack passes diagonally through both the block and the mortar. See W10 in Fig. 2.
- ***C*** → Combined failure: this combines diagonal tension and the shear-splitting failure of the block. See W13 in Fig. 2.
- ***S*** → Sliding failure: the course of blocks moves horizontally due to separation and lack of adhesion between the mortar and the blocks. See W3 in Fig. 3.

**Table 1:** Summary of tested wallettes for both mortar bedding types.

Full					Face				
No.	Shear stress (MPa) (τ)	Shear Modulus (MPa) (G)	Maximum angular strain (mm/mm) (γ)	Failure	No.	Shear stress (MPa) (τ)	Shear Modulus (MPa) (G)	Maximum angular strain (mm/mm) (γ)	Failure
**W1**	0.45	1859	0.0004	**C**	**W1**	0.71	1588	0.0005	**A**
**W2**	0.53	2374	0.0008	**C**	**W2**	0.55	2995	0.0004	**C**
**W3**	0.70	–	–	**C**	**W3**	0.70	1837	0.0007	**S**
**W4**	0.62	2243	0.0008	**C**	**W4**	0.70	2343	0.0010	**S**
**W5**	0.53	1302	0.0004	**C**	**W5**	0.57	1884	0.0009	**A**
**W6**	0.52	1302	0.0005	**A**	**W6**	0.73	1715	0.0008	**A**
**W7**	0.50	1938	0.0010	**C**	**W7**	0.76	–	–	**S**
**W8**	0.41	–	–	**A**	**W8**	0.50	1345	0.0013	**A**
**W9**	0.51	1066	0.0006	**A**	**W9**	0.71	2921	0.0007	**A**
**W10**	0.65	1290	0.0006	**DT**	**W10**	0.52	3174	0.0012	**A**
**W11**	0.69	1634	0.0008	**C**	**W11**	0.68	1569	0.0007	**A**
**W12**	0.35	2419	0.0002	**A**	**W12**	0.61	1491	0.0007	**A**
**W13**	0.52	1452	0.0003	**C**	**W13**	0.59	1583	0.0004	**C**
**W14**	0.72	1962	0.0004	**C**	**W14**	0.43	1625	0.0006	**C**
**W15**	0.71	1895	0.0004	**C**	**W15**	0.43	2358	0.0002	**C**
**W16**	0.72	2893	0.0002	**A**	**W16**	0.34	1739	0.0002	**C**
**W17**	0.49	–	–	**S**	**–**	–	–	–	**–**
**Mean**	0.57	1831	0.00053	–	**Mean**	0.59	2011	0.0007	–
**S.D**	0.11	507	0.00023	–	**S.D**	0.12	578	0.0003	–
**COV**	19.95 %	27.68 %	42.97 %	–	**COV**	20.68 %	28.73 %	45.48 %	–

The failure modes are crucial for understanding the behavior of masonry under shear stress. All failures were documented during testing using photographic images and visual indicators. After testing, each failure was classified according to the NMX-C-464-ONNCCE-2010 [11] standard.

On the other hand, the stress-strain curves provided in this dataset, for both mortar bedding methods, can be used to inform the development of new theoretical models. For instance, by following a methodological approach similar to [23], an analytical model could be derived to describe the shear behavior of HCB masonry for both mortar bedding variants. This could be particularly useful for updating design codes to reflect differences in mechanical performance, ensuring that construction practices in seismic regions are more aligned with safety requirements.

## 4 Experimental Design, Materials and Methods

The experimentation carried out is based on the National Organization for Standardization and Certification of Construction and Building (ONNCCE, by its Spanish acronym) [11,12,[24], [25], [26], [27], [28], [29], [30]], as well as the standard [10]. The methodology and the experimental design were divided into two different phases: the first is the characterization of the masonry component materials (blocks and mortar), and the second is the construction of the masonry wallettes and their diagonal compression testing.

### 4.1 Materials characterization

**Fig. 4:**
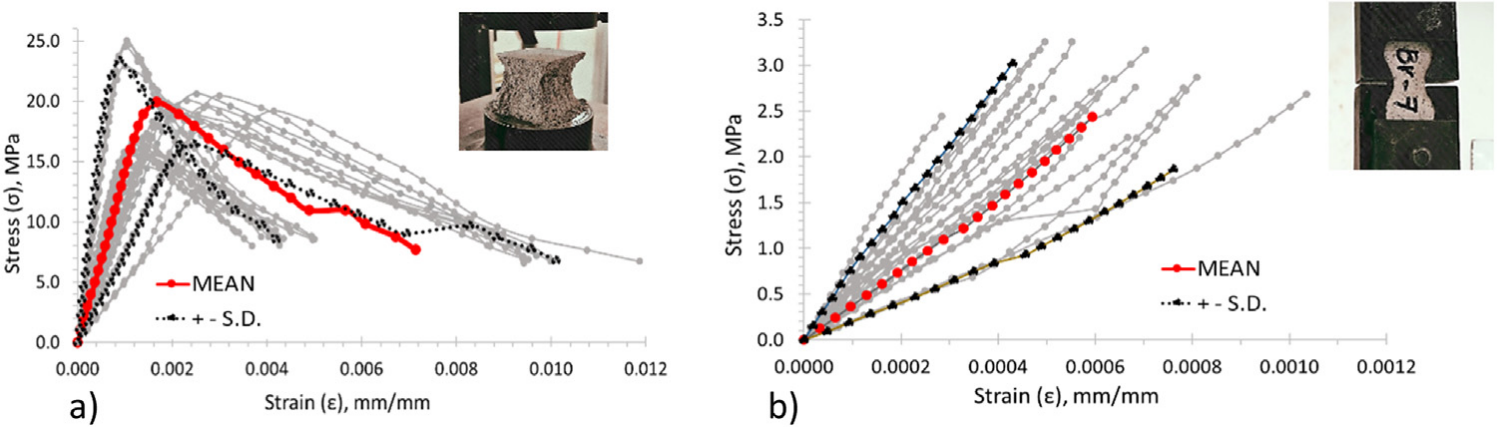
Mortar characterization of: a) Cubes, and b) briquettes.

Mortar and blocks were tested for characterization before the masonry walletes. The materials used for mortar were Portland Cement CPC 30R, water, and sand, with 1:1:6 dosages respectively. The mortar experiments were divided into compressive and tensile strength tests. An Instron 600DX machine was used to test all the mortar specimens. Twenty-seven cubes of 5 cm length were tested under compressive strength, following the specification of standard [19]. Also, twenty-seven briquettes were tested to obtain the tensile behavior of the mortar, following the standard specifications from [19]. The compression and tension tests were carried out by a controlled displacement of 0.005 mm/*sec* and 0.003 mm/s respectively. Strain gauges and the LVDT from the Instron machine were used to measure the strain and displacements under compression and tension. Fig. 4 shows the results obtained from the mortar characterization. The mean maximum compressive and tensile strength was 19.90 MPa and 2.44 MPa correspondingly. The mortar is classified as Type 1 according to the Mexican standard. This information also can be found in the Excel sheet of the dataset.

**Fig. 5:**
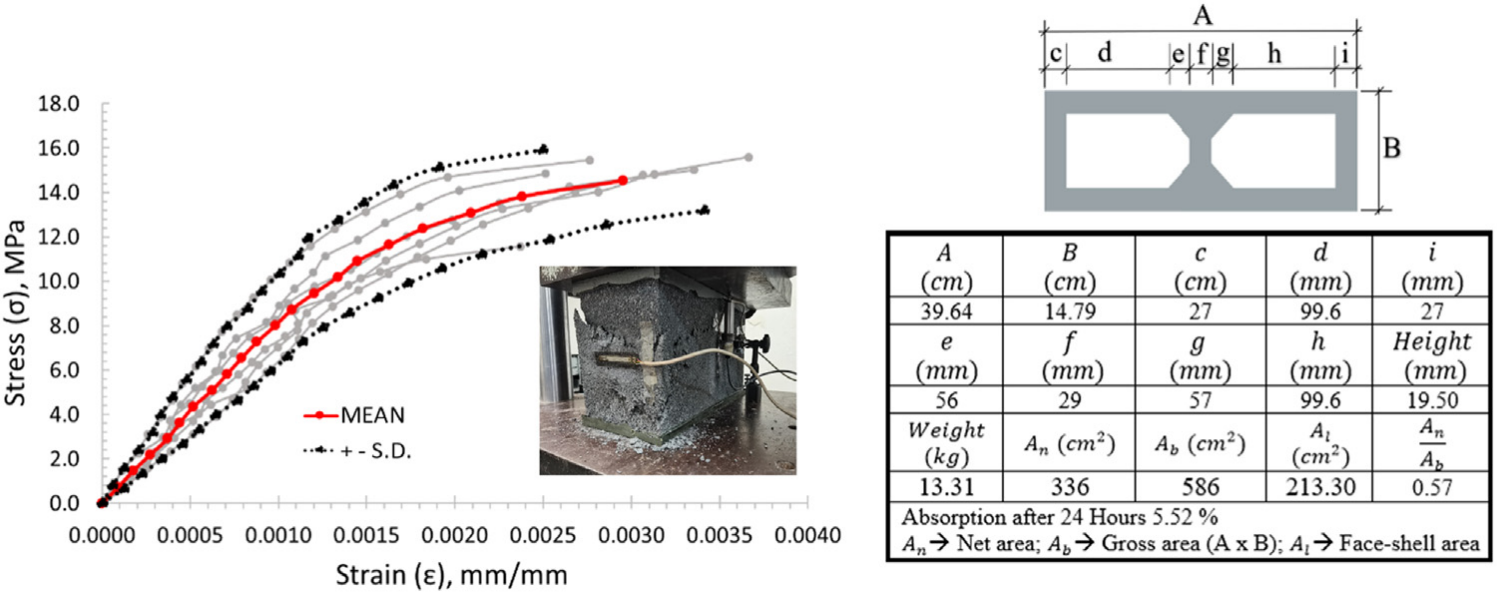
Stress-strain curves of hollow concrete blocks under compressive strength.

On the other hand, hollow concrete blocks manufactured in Nuevo León State, Mexico, were used in the investigation. The blocks have two holes with nominal dimensions of 15 cm × 20 cm × 40 cm (thickness x height x length). Different checks and tests were carried out to characterize the blocks: (1) size measurements using a Vernier caliper and a tape measure [28]; (2) volumetric weight using a scale; (3) absorption was measured using a curing vat [29], a ventilated and temperature controlled oven (105 °C ± 5 °C), and a scale; (4) uniaxial compression test and [27], (5) direct and splitting tensile tests [31]. The average of values measurement in the blocks are shown in Fig. 5 and in the dataset. On the other hand, the mix proportion used in the blocks was 1:4:2.3 (cement/sand/coarse aggregate).

A dosage of 1:2 of sulfur to fly ash was used for capping the blocks in the uniaxial compressive strength test. The Instron machine was employed with load-displacement control of 0.005 mm/s. The average maximum compressive strength of nine blocks was 15 MPa over the net area, as shown in Fig. 5. The characterization of the blocks concerning the tensile strength parameter can be found in references [31,32].

### 4.2 Diagonal compression strength tests

**Fig. 6:**
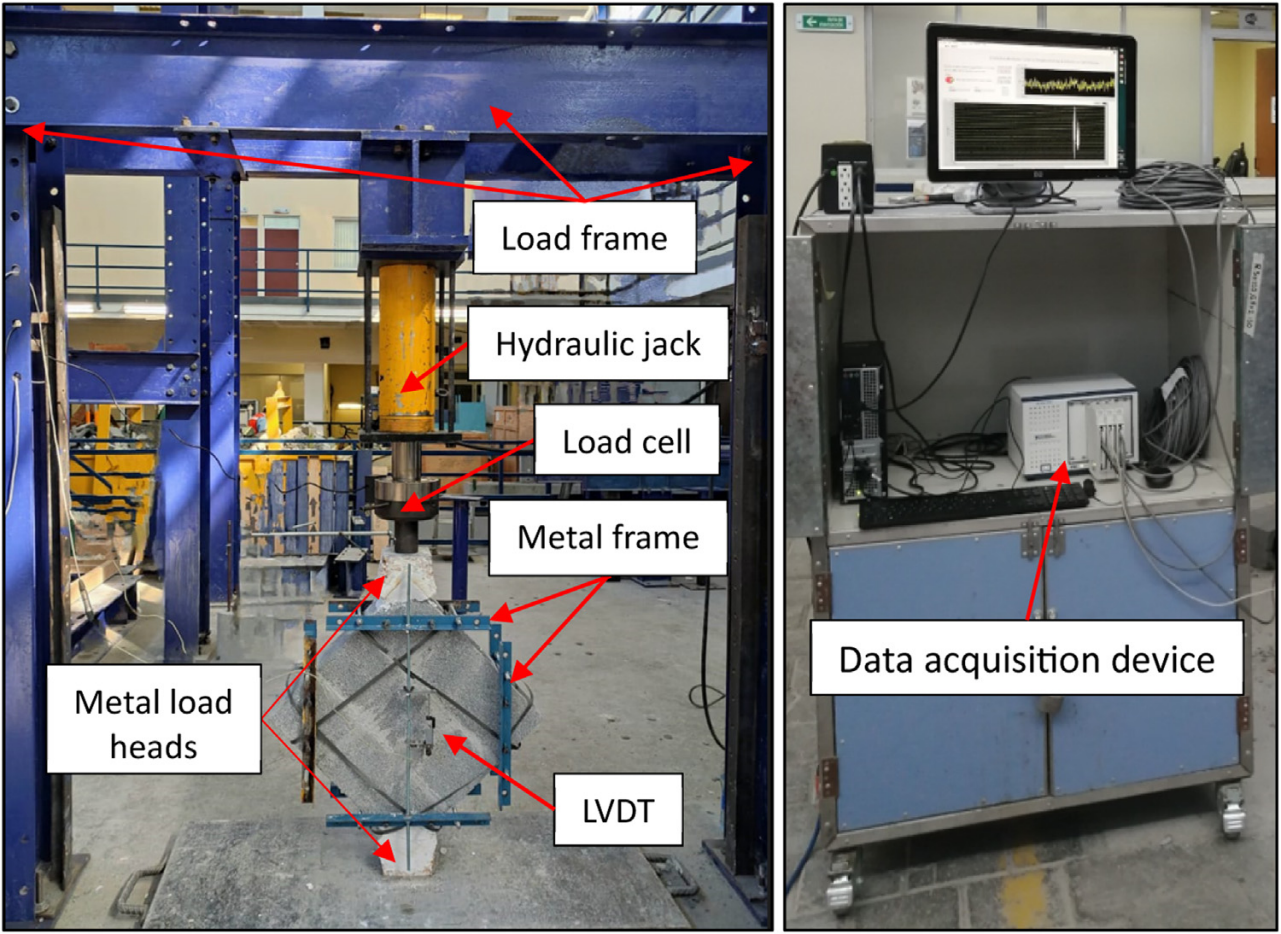
Arrangement and test set up for wallettes.

**Fig. 7:**
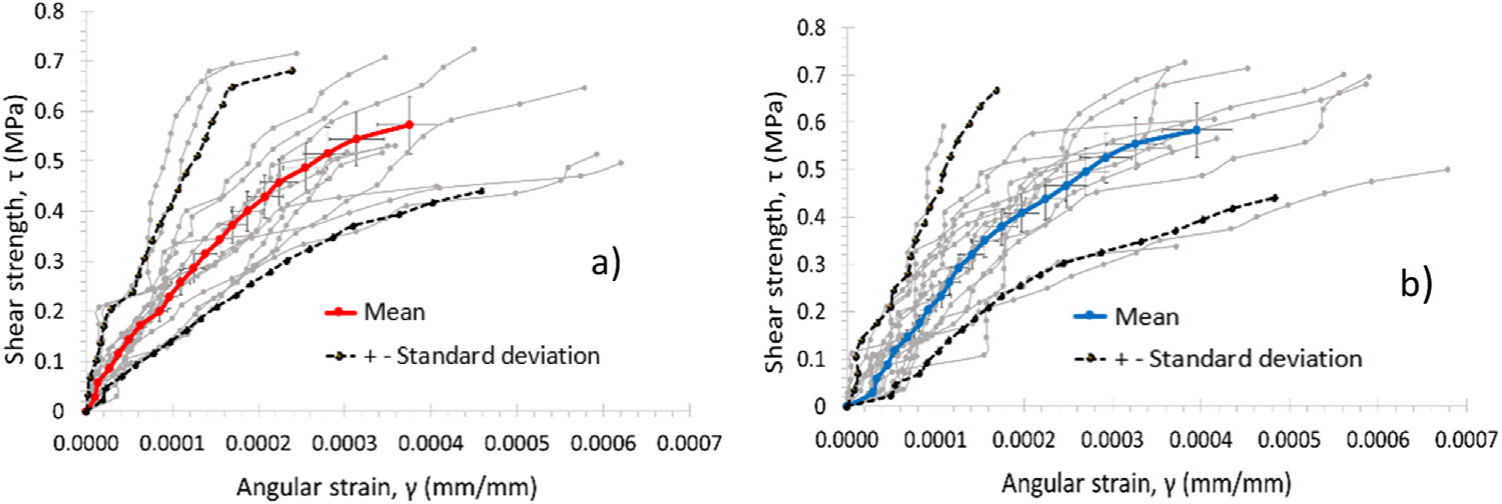
Shear stress vs. angular strain curves measures for wallettes with mortar bedding over the: a) net area (full) and, b) face shell.

Thirty-three wallettes were built with 3 courses of hollow concrete block and tested in this research. Each specimen was prepared by tilting the wallette to a 45-degree angle and securing it with two metal load heads (upper and lower). The metal load heads were joined with a plaster joint and metal frames were used to hold the LVDTs, with two small levels ensuring proper alignment, following the NMX-C-464-ONNCCE-2010 standard, also can be use the ASTM E519/E519M standard [33]. Measurements were taken with a tape measure, and the metal supports were used to hold the transducers. A load frame suitable for the dimensions of the wallettes was constructed. The load was applied using a 100-ton hydraulic jack and load cell. Data collection was conducted through a Wheatstone bridge data acquisition device. The arrangement and test set up used for wallettes is shown in Fig. 6. The dimensions of the load frame, in this case, were 2.40 m width and 2.30 m height, those dimensions can vary depending on the walletes dimensions and the hydraulic jack stroke of the piston. The curves obtained along with the mean and the standard deviation for the walletes can be observed in Fig. 7.

## Limitations

This dataset has several limitations. Firstly, it focuses on specific types of hollow concrete blocks and mortar compositions, which may not be representative of all masonry materials used globally. This restricts the generalizability of the findings to other blocks and mortar types. Secondly, the experimental conditions in a laboratory setting may not perfectly replicate real-world construction environments, where factors such as weather, workmanship, and long-term load effects come into play. Thirdly, the dataset primarily examines shear behavior, leaving other important aspects, such as durability, thermal performance, and acoustic properties of masonry, unexplored. Additionally, the sample size and scope of the experiments might be limited, potentially affecting the statistical robustness of the results.

Moreover, several potential sources of error and uncertainty in the experimental measurements should be considered. Instrument precision may have introduced errors, as even small inaccuracies in the force and displacement sensors could influence the shear strength measurements. Operator error is another factor, as variations in how materials were prepared, tested, or measured might have affected consistency. Additionally, environmental factors during data collection, such as minor fluctuations in temperature or humidity in the lab, could have impacted the performance of both the mortar and the blocks, even though these variables were not the primary focus of the study. While care was taken to ensure repeatability, measurement error remains a possibility due to the inherent variability in construction materials and processes. Finally, variations in construction practices across different regions mean that the findings may not be universally applicable without further localized studies. These limitations should be considered when interpreting the results and applying them to practice or further research.

## Ethics Statement

The authors have read and followed the ethical requirements for publication in Data in Brief and confirm that the current work does not involve human subjects, animal experiments, or any data collected from social media platforms*.*

## CRediT Author Statement

**M Mesa-Lavista**: Conceptualization, Methodology and Writing - Original Draft. **P Romo-Letechipía:** Data Curation, Visualization, and laboratory tests. **J Álvarez-Pérez**: Formal analysis, Investigation and Supervision. **R González-Alcorta**: Funding acquisition, Resources. **J H Chávez-Gómez**: Resources, Writing - Review & Editing and Supervision. **G Fajardo-San-Miguel**: Resources, Writing - Review & Editing and Supervision.

## Data Availability

Mendeley DataDiagonal compressive tests with different mortar bedding (Original data).
